# DECONSTRUCTING THE MODEL-MINORITY MYTH OF U.S. ASIANS: DETERMINANTS AND CONSEQUENCES OF ELDER MISTREATMENT

**DOI:** 10.1093/geroni/igz038.2792

**Published:** 2019-11-08

**Authors:** XinQi Dong, Melissa Simon

**Affiliations:** 1 Rutgers Institute for Health, Health Care Policy and Aging Research, New Brunswick, United States; 2 Northwestern University, Chicago, Illinois, United States

## Abstract

Elder mistreatment (EM) is increasingly recognized as a global health concern. Among U.S. minority and immigrant populations, the social contexts and psychological consequences associated with EM remain poorly understood. Further population-based epidemiological studies using standard EM measures are required to advance the field. To address this gap and to challenge prior assumptions regarding Asian populations, this purpose of this symposium is to improve our understanding of EM epidemiology in an older minority population. Data were drawn from the Population-based Study of Chinese Elderly in Chicago (PINE), a longitudinal, representative, population-based study of 3,157 community-dwelling Chinese older adults in the greater Chicago area. Session 1 will examine the transmission between child mistreatment, intimate partner violence, and EM. Session 2 will take a typology approach to capture the multifaceted family relationships, and will further examine which family typologies were associated with greater likelihood of EM, while which typologies were protective against EM. Session 3 will explore the positive and negative aspects of social support from spouse, family, and friends in relationship to EM subtypes, including psychological, physical, financial and sexual mistreatment, and caregiver neglect. Session 4 will examine the relationship between broad, moderate, and strict definitions of EM and likelihood of experiencing anxiety. Last, Session 5 will explore the differential relationships between EM subtypes and depressive symptoms. In summation, this symposium challenges popular conceptions of the “model minority myth” and aims to increase the practical and clinical relevance of EM epidemiology in community, research, healthcare, and policy settings.

